# Assessment of Inhibitors of Pathogenic Crimean-Congo Hemorrhagic Fever Virus Strains Using Virus-Like Particles

**DOI:** 10.1371/journal.pntd.0004259

**Published:** 2015-12-01

**Authors:** Marko Zivcec, Maureen G. Metcalfe, César G. Albariño, Lisa W. Guerrero, Scott D. Pegan, Christina F. Spiropoulou, Éric Bergeron

**Affiliations:** 1 Viral Special Pathogens Branch, National Center for Emerging and Zoonotic Infectious Diseases, Centers for Disease Control and Prevention, Atlanta, Georgia, United States of America; 2 Infectious Disease Pathology Branch, Division of High Consequence Pathogens and Pathology, National Center for Emerging and Zoonotic Infectious Diseases, Centers for Disease Control and Prevention, Atlanta, Georgia, United States of America; 3 Department of Pharmaceutical and Biomedical Sciences, University of Georgia, Athens, Georgia, United States of America; University of Texas Medical Branch, UNITED STATES

## Abstract

Crimean-Congo hemorrhagic fever (CCHF) is an often lethal, acute inflammatory illness that affects a large geographic area. The disease is caused by infection with CCHF virus (CCHFV), a nairovirus from the *Bunyaviridae* family. Basic research on CCHFV has been severely hampered by biosafety requirements and lack of available strains and molecular tools. We report the development of a CCHF transcription- and entry-competent virus-like particle (tecVLP) system that can be used to study cell entry and viral transcription/replication over a broad dynamic range (~4 orders of magnitude). The tecVLPs are morphologically similar to authentic CCHFV. Incubation of immortalized and primary human cells with tecVLPs results in a strong reporter signal that is sensitive to treatment with neutralizing monoclonal antibodies and by small molecule inhibitors of CCHFV. We used glycoproteins and minigenomes from divergent CCHFV strains to generate tecVLPs, and in doing so, we identified a monoclonal antibody that can prevent cell entry of tecVLPs containing glycoproteins from 3 pathogenic CCHFV strains. In addition, our data suggest that different glycoprotein moieties confer different cellular entry efficiencies, and that glycoproteins from the commonly used strain IbAr10200 have up to 100-fold lower ability to enter primary human cells compared to glycoproteins from pathogenic CCHFV strains.

## Author Summary

The tick-borne Crimean-Congo hemorrhagic fever virus (CCHFV) is the causative agent of a frequently life-threatening disease. CCHFV is present in a wide geographic area with potential for expansion. Moreover, CCHFV segmented genome reassortment leads to new strains with potentially different virulence. Studying CCHFV is highly necessary, but requires dedicated, resource-intensive, high biosafety and security laboratories. In part due to the need for high containment, CCHFV studies have been limited, and developing tools to study CCHFV has been difficult. We report the development of a system that mimics the CCHFV life cycle and produces virus-like particles (VLPs) that are similar to CCHFV in cell culture, but do not form infectious CCHFV and therefore do not require the use of special laboratories. We generated VLPs representing several pathogenic CCHFV strains with robust reporter signal activity. This allows VLPs to be used in testing cell entry inhibitors against a wide array of CCHFV strains. In addition, VLPs can be used in a variety of cell lines and in cells directly isolated from humans. Our results also suggest that the CCHFV strain IbAr10200, which is commonly used in the laboratory, may not accurately reflect the activity of circulating pathogenic CCHFV strains, as the surface glycoproteins of IbAr10200 confer reduced entry efficiency of VLP into cells derived directly from humans. In addition, we show that drugs with proven anti-CCHFV properties inhibit VLP activity, and identify a monoclonal antibody that prevents cell entry of VLP made using glycoprotein genes from different, pathogenic CCHFV strains.

## Introduction

Crimean-Congo hemorrhagic fever (CCHF) is a rapidly progressing inflammatory illness with high case fatality rates and a vast endemic area [1–6]. The etiological agent, CCHF virus (CCHFV), is a tri-segmented virus belonging to the *Nairovirus* genus of the *Bunyaviridae* family; it is primarily maintained in and transmitted by *Hyalomma* species ticks [1,5,6]. Human infection is usually associated with tick bites or by unprotected contact with bodily fluids of infected animals or humans.

Subclinical and mild cases of CCHFV infection usually consist of non-specific “flu-like” symptoms (fever, vomiting, and diarrhea), and are self-resolving. Severe CCHFV infection progresses to CCHF, which is characterized by petechiae, ecchymosis, epistaxis, gingival hemorrhage, and, frequently, gastrointestinal and cerebral hemorrhage [1,7,8]. Case fatality rates of CCHF vary among outbreaks and potentially among strains of CCHFV, but are approximated to 30% of clinical cases [9,10]. The broad endemic region and high fatality rate of CCHF necessitate further research into the biology of CCHFV and development of effective prophylactic and therapeutic options to treat CCHFV infections for mitigating the negative public health impact of this pathogen.

Basic research on CCHFV and the development of CCHF therapies and prophylaxes have been severely hampered by a number of factors. Safe handling of CCHFV requires high-containment facilities (biosafety level 3 (BSL-3) and BSL-4 facilities in endemic and non-endemic areas, respectively [9]). In addition, while CCHFV strains are highly variable in nature, laboratory strain availability is limited; the majority of basic research uses strain IbAr10200, which has unknown pathogenicity in humans. Furthermore, due to technical difficulties in engineering recombinant CCHFV and pseudo-typing CCHFV glycoproteins onto other viruses, few and very limited reporter systems of CCHFV are available [11–14].

The major viral components of CCHFV particles consist of the viral genome and proteins. CCHFV, like all *Bunyaviridae* members, has a tri-segmented, negative sense RNA genome. The 3 segments, named small (S), medium (M), and large (L), encode the viral nucleocapsid protein (NP), the glycoprotein precursor (GPC), and the viral polymerase (L), respectively. Each of the CCHFV genomic segments consist of a coding region flanked by 5′ and 3′ non-coding regions (NCRs). The NCRs are sufficient to initiate viral transcription, replication, encapsidation of RNA by NP and L, and packaging the RNA into viral particles [12,15]. An infectious CCHFV particle consists of at least all 3 viral RNA segments bound to NP and L (ribonucleoprotein complexes, RNPs) that are encapsulated by a lipid membrane containing the mature glycoproteins Gn and Gc, which are generated by post-translational processing of the GPC. Unlike many other negative-strand RNA viruses, bunyaviruses do not encode a separate matrix protein responsible for driving virus formation and incorporating RNPs into nascent viral particles. Rather, these processes are thought to be mediated by interaction between the RNPs and Gn and/or Gc [16–19]. Furthermore, as Gn and Gc mediate CCHFV entry into cells, these proteins are thought to be the major targets of host neutralizing antibody responses. Presumably due to this immunologic pressure, rapid mutation and frequent reassortment of the M segment have been reported in phylogenetic studies of CCHFV [20,21].

CCHFV reassortment occurs when the same cell is co-infected with at least 2 CCHFV strains. In order for reassortment to occur, the genomic NCRs and viral proteins must be compatible (i.e., the strains must possess both RNA-protein and protein-protein compatibility). How interaction of NCRs with RNPs and structural glycoproteins influences the reassortment observed in CCHFV is unknown [20].

To accelerate studies of CCHFV, we optimized a virus-like particle (VLP) system for use in a BSL-2 setting. The VLPs are transcription- and entry-competent VLPs (tecVLP), do not produce infectious CCHFV, and are morphologically similar to CCHFV. We show that the tecVLP system is suitable for addressing the following points: (1) screening antivirals; (2) testing potency of monoclonal antibodies against divergent CCHFV strains; and (3) identifying potential molecular determinants of CCHFV reassortment, such as compatibility between NCRs and glycoproteins from various CCHFV strains. Unlike previous CCHFV reporter systems [12–15], transfection or other pre-treatment of target cell lines is not required for tecVLP activity, so diverse cell lines and human primary cells may be used in tecVLP assays without special modification. This molecular tool may therefore be used in elucidating important aspects of CCHFV biology, in high-throughput screening, and in developing effective clinical countermeasures against CCHFV.

## Materials and Methods

### Biosafety and safety testing

Trained personnel performed all procedures involving potentially infectious agents (work with infectious CCHFV and initial safety experiments with tecVLPs) in a BSL-4 facility according to standard operating procedures approved by the institutional biosafety committee. Other procedures were carried out under BSL-2 conditions.

### Ethics statement

The use of human blood products was approved by Emory University Institutional Review Board (IRB reference IRB00045947). Under this protocol, no donor personal information was provided, and informed consent was given. All adult blood donors provided informed consent, and a parent or guardian of any child participant provided informed consent on the child’s behalf.

### Cell lines

HuH7 cells (obtained from Apath LLC, Brooklyn, NY, USA) were propagated in Dulbecco’s modified Eagle’s medium (DMEM) supplemented with 10% fetal bovine serum (FBS), 1% non-essential amino acids, and 1% penicillin/streptomycin (all from Life Technologies, Grand Island, NY, USA). BSR-T7 cells (a kind gift from K.K. Conzelmann, Ludwig-Maximilians-Universität, Munich, Germany), which constitutively express T7 polymerase, were propagated in DMEM supplemented with 5% FBS, 1% sodium pyruvate, 400 ng/mL G418, and 1% penicillin/streptomycin. A549 cells (ATCC, Manassas, VA, USA) and SW-13 cells (a kind gift from P. Leyssen, Rega Instituut KU, Leuven, Belgium) were both propagated in DMEM supplemented with 10% FBS, 1% sodium pyruvate, and 1% penicillin/streptomycin. All cells were grown in a humidified 37°C, 5% CO_2_ incubator.

Peripheral blood mononuclear cell (PBMC) pheresis products were obtained from a single healthy human donor at Emory University hospital (Atlanta, GA, USA). PBMC were purified from whole blood pheresis products using Ficoll-Paque (GE Healthcare, Atlanta, GA, USA) according to manufacturer's instructions. Monocytes were isolated from the purified PBMC using the human Monocyte Isolation Kit II (Miltenyi Biotec Inc., San Diego, CA, USA) according to manufacturer’s instructions, and frozen in liquid nitrogen until use. Monocytes were plated in 96-well cell culture plates at a density of 2.5 × 10^5^ cells/cm^2^ in RPMI media supplemented with 10% FBS and 1% penicillin/streptomycin, and cultured for 10 days to allow differentiation into monocyte-derived macrophages. RPMI media was replaced every 2–3 days. GM05659 cells (apparently healthy, non-fetal human fibroblasts from chest skin, obtained from Coriell Institute, Camden, NJ, USA) were cultured in DMEM supplemented with 10% FBS, 1% sodium pyruvate, and 1% penicillin/streptomycin, and grown in a humidified 37°C, 5% CO_2_ incubator.

### Sequencing the M segment of novel CCHFV strains

Sequences of the M genomic segments of the novel CCHFV isolates were generated as previously described [21]. Reverse transcription PCR (RT-PCR) amplification products of the M segments were analyzed by Sanger sequencing and uploaded to GenBank.

### Helper plasmid construction

CCHFV helper plasmid genes were synthesized from GenBank sequences by GenScript USA Inc. (Piscataway, NJ, USA). The CCHFV reference strain IbAr10200 L polymerase gene was codon-optimized and cloned into the mammalian expression plasmid pCAGGS-LCK to produce helper plasmid pC-L, as previously described [11]. GPC genes from CCHFV strains IbAr10200 (reference CCHFV strain), Afg2990 (human lethal case), and from CCHFV isolated from human patient samples collected in Oman and Turkey were codon-optimized, synthesized by GenScript, and cloned into the mammalian expression plasmid pCAGGS (pC). These constructs were named pC-GPC-IbAr, pC-GPC-Afg, pC-GPC-Oman, and pC-GPC-Turk. Both pCAGGS and pCAGGS-LCK (both abbreviated to pC) have the same mammalian promoter for expression, but the pCAGGS-LCK is a low copy plasmid in bacteria to facilitate efficient bacterial production of the unstable pC-L construct. The previously described pC-NP plasmid was used to express NP from CCHFV strain IbAr10200 [11,12]. The previously described T7 polymerase plasmid pC-T7 was also used [11].

### Minigenome plasmid construction

**Fig 1:**
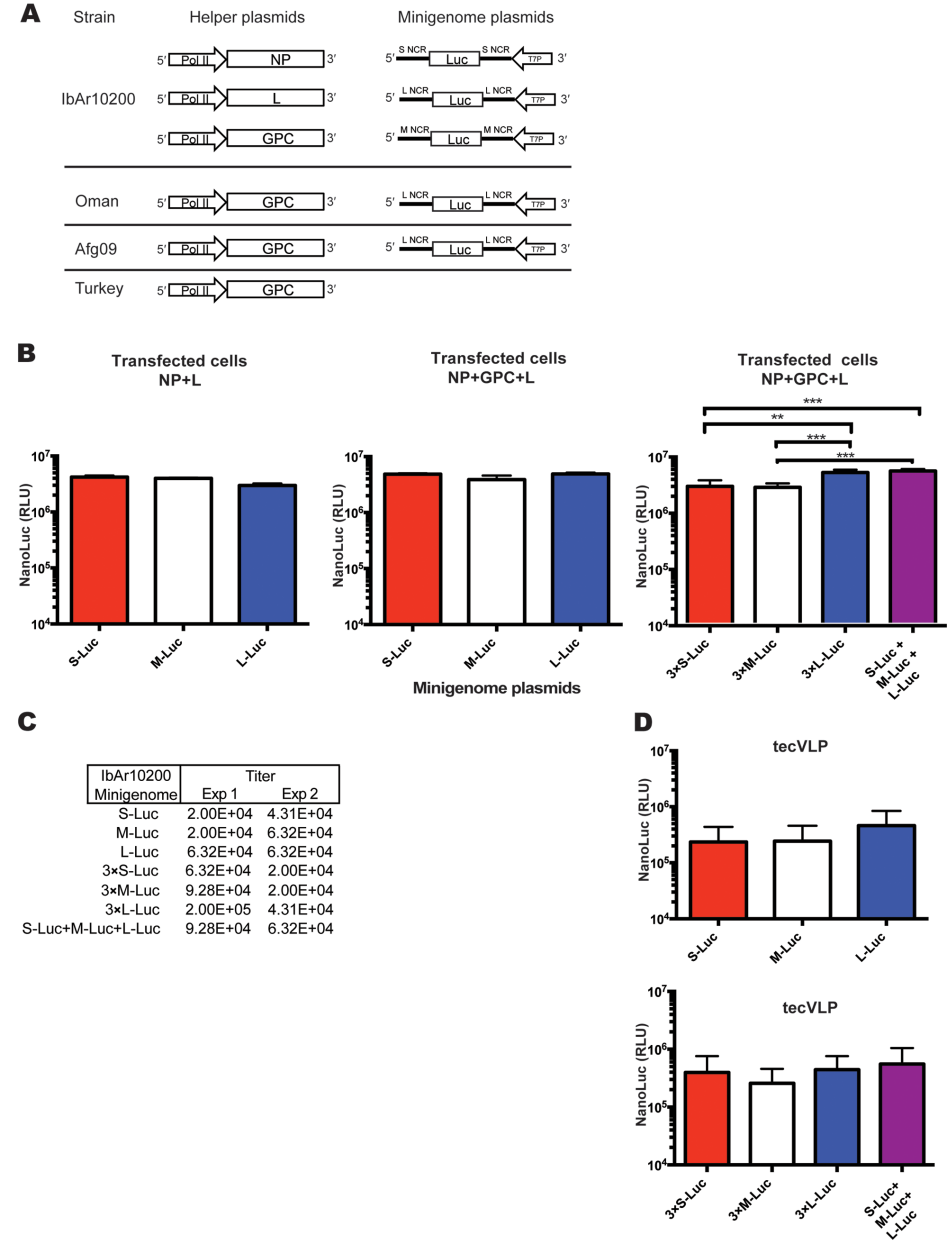
Organization and signal expression of the Crimean-Congo hemorrhagic fever virus (CCHFV) minigenome and support plasmids in transfected cells, and in cells treated with transcription- and entry-competent virus-like particles (tecVLP). (A) Basic organization of CCHFV helper plasmids and minigenomes used in these experiments. Helper plasmids encoding the nucleocapsid protein (NP), the viral RNA-dependent RNA-polymerase (L), and glycoprotein precursor (GPC) are under the control of the chicken β-actin polymerase II (pol II) promoter. Replication machinery genes (NP and L) are from the CCHFV strain IbAr10200, and the surface GPC are from IbAr10200, Oman, Turkey, or Afg09 CCHFV strains, as indicated. The minigenome plasmids encode the NanoLuc (Luc) reporter gene in reverse, or non-coding, sense; NanoLuc is flanked by non-coding regions (NCR) from one of the 3 CCHFV genomic segments, S, M, or L. Minigenomes of all 3 segment NCRs from strain IbAr10200, and only the L segment NCR from strains Afg09 and Oman, were used in these experiments. (B) Absolute NanoLuc signal levels, in relative light units (RLU), following transfection of BSR-T7 cells with minigenome and replication plasmids (NP + L) or minigenome and tecVLP assembly plasmids (L + NP + GPC). Data are reported as standard error of the mean. ** p < 0.01, ***p < 0.001 (average of 2 experiments). (C) Titration experiments from 2 tecVLP production experiments passaged into SW-13 cells. S, M, and L NCR minigenomes are shown. (D) NanoLuc signal (in RLU) in SW-13 cells treated with supernatants from BSR-T7 cells transfected with tecVLP assembly plasmids (average of 2 experiments). NanoLuc signal data in tecVLP-treated cells are presented as absolute RLU values which are calculated as signal in SW-13 cells treated with entry-competent VLPs (i.e., containing NP, L, and GPC) minus signal in SW-13 cells treated with VLPs containing only NP and L. All data are reported as standard error of the mean.

Minigenomes encoding the NanoLuc luciferase gene (Promega, Madison, WI, USA) sequence flanked by S, M, or L NCRs from CCHFV strain IbAr10200, or with L NCRs from the CCHFV samples from Oman or Afg2990, were gene-synthesized (IDT, Coralville, IA, USA). The minigenomes were cloned into pSMART-LCK plasmid (Lucigen, Middleton, WI, USA). The resulting plasmids (pS-Luc, pM-Luc, pL-Luc, pOmanL-Luc, and pAfgL-Luc) expressed viral-sense (i.e., negative or non-coding sense) RNA fragments containing CCHFV recognition signals under the control of a T7 promoter, and containing one extra G at the 5′ end of the transcripts to enhance transcription by the T7 polymerase (Fig 1A).

### Plasmid transfections for tecVLP production

BSR-T7 cells were seeded in multi-well plates overnight and transfected with combinations of minigenome and helper plasmids. Plasmids were transfected using TransIT-LT1 Transfection Reagent according to manufacturer’s recommendations (Mirus Bio LLC, Madison, WI, USA). Helper plasmids were transfected in a weight ratio of pC-NP:pC-GPC:pC-L:pC-T7:minigenome of 4:10:2:4:1 unless otherwise noted, and if a helper plasmid was omitted, it was replaced with an equal weight of empty pC. The ratio of minigenome to helper plasmid was kept constant. The total amount of transfected DNA varied according to the size of the culture plate well: a 6-well plate was transfected with 5 μg of total DNA per well, while a 24-well plate was transfected with 1 μg of total DNA per well. To minimize carry-over of plasmids to the subsequent passage, transfection media was removed ~16–18 h post transfection and replaced with fresh media. Cell lysates, or supernatants for tecVLP passaging, were collected 3 days post transfection.

### Luciferase assays

NanoLuc signal was assayed in BSR-T7 cells transfected with CCHFV minigenome plasmids and in A549, BSR-T7, GM05659, HuH7, monocyte-derived macrophages, and SW-13 incubated with tecVLPs. Briefly, culture media was removed from the cells, and the cells were then washed once with PBS, and either frozen at -20°C or lysed by incubating for 30–45 min in passive lysis buffer (Promega) at room temperature. 20 μL of cell lysate was removed and assayed with Nano-Glo Luciferase Assay System (Promega) to detect NanoLuc signal. All luminescence readings were carried out in opaque, white 96-well plates using the Synergy 4 instrument (BioTek Instruments Inc., Winooski, VT, USA).

### tecVLP safety testing

BSR-T7 cells were transfected with plasmids for tecVLP rescue as described in “Plasmid transfections for VLP production” section, or with pC as a mock transfection control. In parallel, supernatants from tecVLP-producing or mock-transfected BSR-T7 cells, and CCHFV strain IbAr10200 viral stock (SW-13 cell supernatants), were clarified by low speed centrifugation (1500 × g for 10 min). SW-13 cells were seeded at ≥ 90% confluence and allowed to adhere overnight. The media was removed from the SW-13 cells, and the cells were incubated with supernatants from mock-, tecVLP-, or CCHFV-treated cells for 2 h at 37°C. After incubation, the supernatants were removed and replaced with fresh media.

Two days post incubation, cells were fixed with 10% formalin-buffered solution and permeabilized with Triton-X100. Presence of CCHFV antigens was detected by incubation with CCHFV hyperimmune mouse ascetic fluid (HMAF, made in-house at CDC, Atlanta, GA, USA) or monoclonal antibody (mAb) 13G8 (BEI Resources, Manassas, VA, USA) followed by incubation with goat anti-mouse Alexa 488-conjugated antibody (Life Technologies). Images were captured with a TCS SP5 confocal microscope (Leica Microsystems, Buffalo Grove, IL, USA).

### tecVLP passaging

BSR-T7, HuH7, SW-13, A549, and GM05659 cells were seeded at ≥ 70% confluence and allowed to adhere overnight. Plates of primary human macrophages were seeded at 2.5 × 10^5^ cells/cm^2^, and allowed to differentiate for 10 days. For all cells, media were removed and cells incubated with tecVLP-containing supernatants from transfected BSR-T7 cells for at least 2–3 h at 37°C. After incubation, the tecVLP-containing supernatants were removed, and the cells were washed 3 times with sterile PBS or RPMI media prior to adding fresh media. The cells were incubated overnight at 37°C to allow biosynthesis of NanoLuc in the cells. Following the incubation period, the cell medium was removed and the cells were washed once more with PBS; the cells were then incubated with passive lysis buffer for NanoLuc assays, or frozen at -20°C.

### tecVLP titration

tecVLP titration was performed on 10-fold serial dilutions (prepared in DMEM) of transfected cell supernatants, using a TCID_50_ assay on SW-13 cells in 96-well plates. Plates were incubated with the supernatants overnight at 37°C, and were then treated with passive lysis buffer for NanoLuc assays, or frozen at -20°C. Wells that displayed NanoLuc signal at least 3 standard deviations above background levels were considered positive for tecVLP signal. tecVLP concentrations were calculated using the Reed and Muench formula [22], and expressed as TCID_50_ per mL of stock.

### Inhibitors of entry and transcription of tecVLPs

CCHFV IbAr10200 PreGn- and Gc-specific mAbs (13G8 for PreGn; 11E7 and 12A9 for Gc), and CCHFV IbAr10200 NP-specific mAb (9D5) were obtained from BEI Resources. mAbs were diluted in DMEM supplemented with 5% FBS to equal starting concentrations, and further diluted in a 2-fold dilution series (concentration range 1 × 10^1^ to 8 × 10^−2^ μg/mL, or ~1:100 to 1:12800-fold dilution). The mAb dilutions were mixed with an equal volume of tecVLP-containing supernatant and incubated for 1–2 h at 37°C. The mixture was then applied to confluent monolayers of SW-13 cells, and incubated as outlined in tecVLP passaging.

Ribavirin and chloroquine sulfate were obtained from U.S. Pharmacopeia (Rockville, MD, USA). The inhibitors were dissolved in DMSO (Sigma-Aldrich, St. Louis, MO, USA) and diluted in OptiMem (Life Technologies) to starting concentrations, and further diluted in a 2-fold dilution series (final concentration range of 400 μM to 3 μM for ribavirin, and 500 μM to 3.9 μM for chloroquine). The diluted drugs were added to monolayers of SW-13 cells, and cells were incubated for 15–20 min at 37°C. After incubation, equal volume of tecVLP-containing supernatants were added to the drug mixtures, and cells were incubated for 1–2 h at 37°C. The inocula were removed and the cells washed 3 times with sterile PBS prior to addition of fresh media with the same compound concentration; cells were then incubated overnight as described in tecVLP passaging.

Cell viability was determined concurrently with tecVLP signal inhibition experiments, but on only compound-treated cells. Viability was determined using the CellTiter-Glo Luminescent Cell Viability Assay (Promega) according to manufacturer’s instructions.

### tecVLP concentration

BSR-T7 cells in 6-well plates were transfected with plasmids necessary for the production of tecVLP as described in the “Plasmid transfections for tecVLP production” section above. After 3 days, tecVLP-containing cell supernatants were removed, clarified by centrifugation (5–10 min at 1500 × g), filtered through 0.22 μm pore size filters (EMD-Millipore, Billerica, MA, USA), and concentrated in 100 kDa cutoff Centricon Plus-70 centrifugal filter units (EMD-Millipore) to 5–100-fold concentration.

### Electron microscopy

Concentrated tecVLP-containing supernatants were fixed by incubation with an equal volume of 5% paraformaldehyde and stored at 4°C until use. The concentrated, fixed tecVLP samples were processed as follows. First, 2 μL of each sample was pipetted onto a 300-mesh formvar/carbon-coated nickel grid (EMS, Hatfield, PA, USA), and the sample was incubated overnight at 4°C. The samples were then blotted, rinsed with bacitracin (50 μg/mL) [23], blotted, negatively stained with 5% ammonium molybdate (pH 6.9) and 0.1% (w/v) trehalose, and blotted a final time [24]. The grid was examined using a Tecnai BioTwin transmission electron microscope (FEI Company, Hillsboro, OR, USA) operating at 120 kV, and images were captured with a 2K × 2K camera (AMT Corp., Woburn, MA, USA).

### Western blotting

Concentrated tecVLP-containing supernatants or pC-GPC-transfected BSR-T7 cell lysates were incubated with NuPAGE LDS Sample Buffer for 10 min at 70°C prior to loading onto NuPAGE Novex 3–8% tris-acetate protein gels (all from Life Technologies). The gels were run at a constant 200 V for 45 min, and transferred onto nitrocellulose membranes using the iBlot instrument (Life Technologies) according to manufacturer’s instructions. The membranes were incubated overnight at 4°C with anti-N mAb 9D5 (BEI Resources; diluted 1:1000), anti-Gn polyclonal rabbit sera [25] (a kind gift from A. Mirazimi, Karolinska Institutet, Sweden; diluted 1:500), or anti-Gc mAb 7E11 (BEI Resources; diluted 1:1000). Signals were detected with Fast Western Blot Kits mouse or rabbit SuperSignal West Dura (Thermo Fisher Scientific Inc., Waltham, MA, USA) according to manufacturer’s recommendations.

### Phylogenetic analyses

Full-length CCHFV M gene sequences were translated and aligned using the ClustalW algorithm, and phylogenetic trees were constructed using Mega (Biodesign Institute, Tempe, AZ, USA) [26] via the Jukes-Cantor neighbor-joining method with bootstrapping to 10000 iterations.

### Statistical analyses

Analyses were done using one-way or two-way analyses of variance (ANOVA) with Tukey’s multiple comparisons test. The analyses were performed using GraphPad Prism version 6.00 for Mac OS X (GraphPad Software Inc., La Jolla, CA, USA). For inhibitor dilutions, GraphPad Prism was used to fit a 4-parameter equation to semilog plots of the concentration-response data. The plot was used to interpolate the concentration of compound that inhibited 50% of the NanoLuc signal in target cells (EC_50_). The 50% cytotoxic concentration (CC_50_) was derived using luciferase signal levels from inhibitor-treated cells. The selectivity index (SI) was calculated by dividing the CC_50_ by the EC_50_.

### Accession numbers

Helper plasmid genes were based on published and novel CCHFV, and T7 polymerase sequences. Replication machinery plasmids relied on published reference strain IbAr10200 gene sequences (NP gene accession no. NC_005302; L gene accession no. AY389508). GPC helper plasmid gene sequences were based on published sequences of CCHFV strains IbAr10200 (accession no. NC_005300) and Afg2990 (accession no. HM452306.1), or on sequences of novel CCHFV isolates (Oman-811466, accession no. KR864901; Turkey-810473, accession no. KR864902). T7 polymerase helper plasmid was based on a published gene sequence (accession no. M38308).

Minigenomes sequences were based on a published NanoLuc luciferase gene sequence (accession no. JQ437370;) flanked by CCHFV strain IbAr10200 S, M, or L NCRs (S accession no. NC_005302; M accession no. NC_005300; L accession no. AY389508), or by L NCRs from strains Oman (accession no. DQ211619) or Afg2990 (accession no. HM452307).

## Results

### tecVLPs are efficiently produced and are morphologically similar to CCHFV

While the tecVLP system reported here is based on similar premises as previous systems [12,13], it incorporates 2 modifications: the reporter is NanoLuc, a smaller protein that generates a brighter signal than many other luciferases; and the CCHFV L [11] and GPC helper plasmid sequences are codon-optimized.

Because previous reports have indicated differences in the efficiency of transcription, replication and/or packaging of the 3 genomic CCHFV segments [12,13] we first tested which segment NCR was transcribed most efficiently in our system. In order to assess the reporter signal produced, we transfected cells with IbAr10200 helper plasmids and minigenomes containing S, M, and L genome segment NCRs (Fig 1A). The resulting NanoLuc signal was similar regardless of segment used, suggesting no differences in transcription or replication of the 3 genomic segment NCRs (Fig 1B). To determine whether using minigenomes of all 3 segments together would boost the overall signal, equal amounts of S-Luc, M-Luc, and L-Luc were transfected into BSR-T7 together. We then compared the resulting signal to that in cells transfected with triple the amount of individual minigenomes (3 × S-Luc, 3 × M-Luc, or 3 × L-Luc). The highest signal levels were seen when using 3 × L-Luc or S-Luc + M-Luc + L-Luc, (Fig 1B). While there was a difference in signal levels in cells transfected with different minigenomes, the titers of tecVLPs (~2–6 ×10^4^ TCID_50_/mL; Fig 1C) were not apparently different. Likewise, when the tecVLPs resulting from minigenome transfections were passaged to SW-13 cells, the NanoLuc signal levels generated by tecVLPs were similar regardless of minigenome used (Fig 1D). To be consistent in subsequent experiments, we used the L-Luc minigenomes at the original concentration.

Due to safety concerns, we also verified that cells treated with tecVLPs do not release infectious virus. SW-13 cells were treated with infectious CCHFV, tecVLP (in supernatants from transfected BSR-T7 cells), or supernatants of untransfected BSR-T7 cells (negative control). Following 2 days of incubation, the SW-13 cells were stained by standard immunofluorescent assay. The cells inoculated with CCHFV had readily detectable viral antigens (S1A Fig), while cells incubated with tecVLPs or control supernatants did not (S1B and S1C Fig). In addition, SW-13 cells incubated with tecVLPs did not produce new tecVLPs, as supernatants of SW-13 cells treated with tecVLPs did not result in production of NanoLuc signal when passaged onto naïve cells (S1D Fig). Therefore, we concluded that the tecVLPs are incapable of spreading.

**Fig 2:**
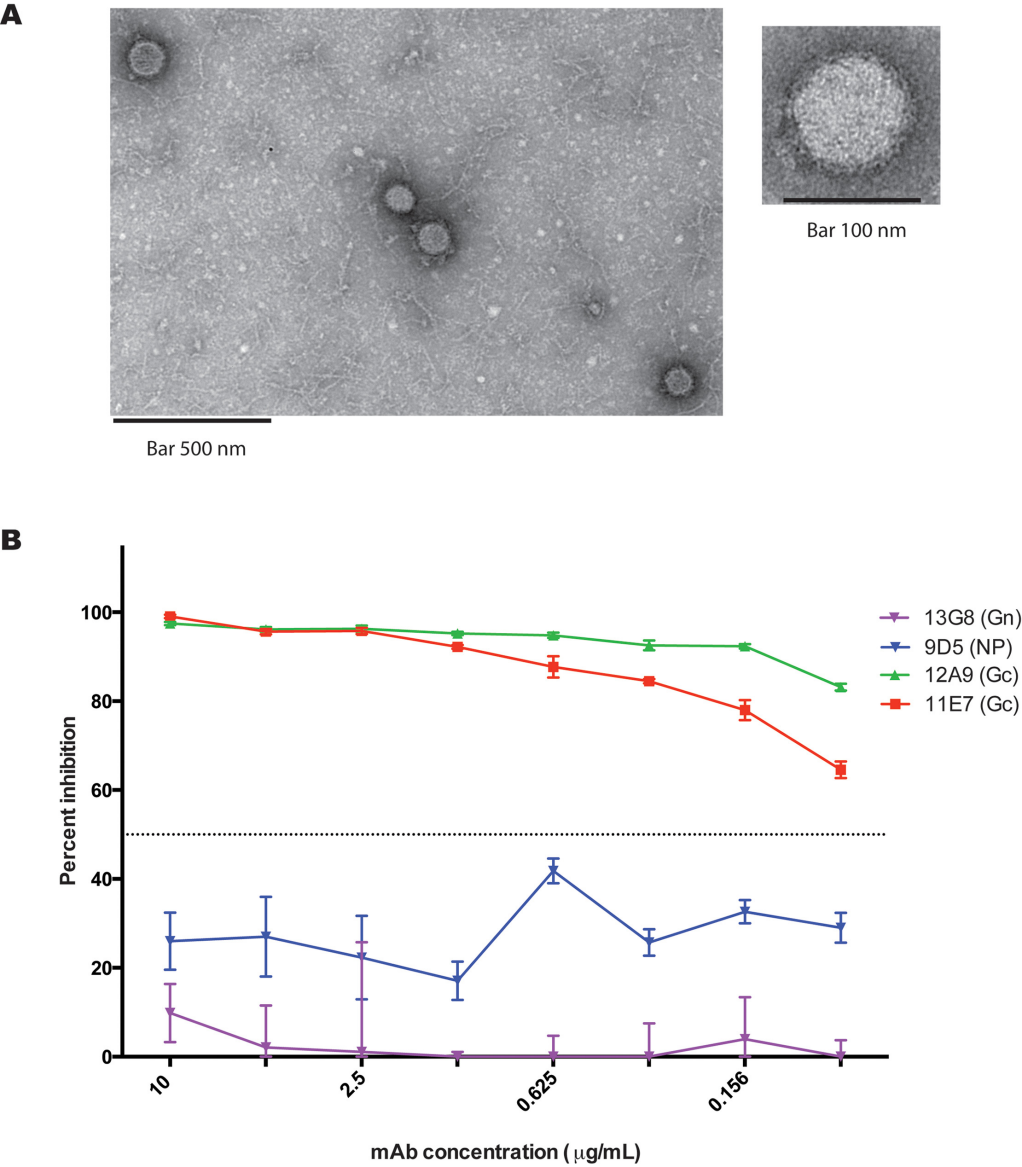
CCHFV tecVLP morphology and glycoprotein-mediated entry. (A) Representative electron microscopy images of tecVLP. (B) Neutralization of tecVLP entry into SW-13 cells by previously reported monoclonal antibodies (mAbs). 11E7 and 12A9 are CCHFV neutralizing antibodies targeting the glycoprotein Gc; 13G8 is a non-neutralizing mAb targeting the immature glycoprotein PreGn; 9D5 targets the NP protein. Data are displayed as standard error of the mean (average of 2 experiments).

To compare tecVLP morphology and cell entry to those of authentic CCHFV, we used electron microscopy and a neutralization assay, respectively. Electron microscopy showed that tecVLPs were morphologically consistent with CCHFV and other bunyaviruses, and were relatively uniform in size (94 ± 3 nm, n = 13, from 5 fields; average ± standard error of the mean, Fig 2A). Furthermore, tecVLPs were neutralized by previously reported neutralizing monoclonal antibodies [27] targeting IbAr10200 strain glycoprotein Gc (11E7 and 12A9), but not by mAbs targeting strain IbAr10200 NP (9D5) or the glycoprotein PreGn (13G8) (Fig 2B). These data suggest that CCHFV IbAr10200 tecVLPs are, from a structural perspective, consistent with bona fide CCHFV particles.

### Oman and Afg09 NCR minigenomes are efficiently transcribed, replicated, and packaged into tecVLPs by IbAr10200 proteins

**Fig 3:**
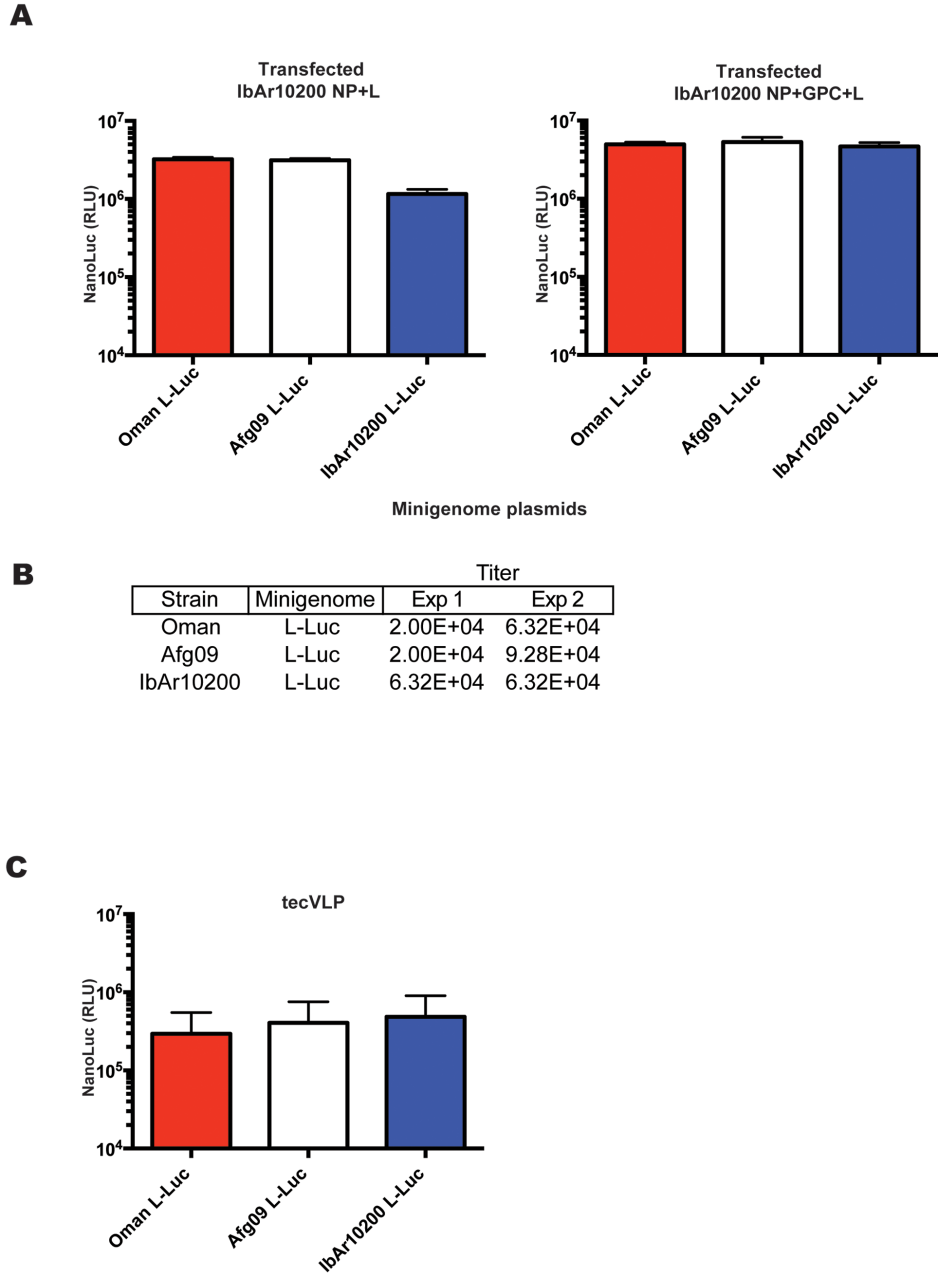
CCHFV strain IbAr10200 replication machinery does not significantly discriminate against minigenomes from other CCHFV strains. (A) Absolute NanoLuc signal in relative light units (RLU) in BSR-T7 cells transfected with IbAr10200 NP, L, and GPC plasmids, and with minigenomes encoding L NCR of other CCHFV strains, as indicated. Data are reported as standard error of the mean (n = 8 from 2 experiments). (B) TCID_50_ determination in SW-13 cells treated with supernatants from cells in (A). Results from 2 experiments are shown. (C) NanoLuc signal in SW-13 cells treated as in (B) (n = 8 from 2 experiments). NanoLuc signal data in VLP-treated cells are presented as absolute RLU values which are calculated as signal in SW-13 cells treated with entry-competent VLPs (i.e., containing NP, L, and GPC) minus signal in SW-13 cells treated with VLPs containing only NP, L and the corresponding minigenome. All data are reported as standard error of the mean.

Due to the sequence variability between the NCRs of different CCHFV strains, we expected differences in the efficiency with which viral sense RNAs are recognized and transcribed or packaged into particles by IbAr10200 proteins. To study the role of NCR sequences in tecVLP production, L NCR minigenomes from strains isolated from severe human cases of CCHFV (Oman and Afg09) were expressed in the tecVLP system. tecVLPs were generated using minigenome plasmids containing Oman or Afg09 L NCRs flanking NanoLuc, and the same IbAr10200 helper plasmids (NP + GPC + L) as used previously (Fig 1A). The results show that NCRs from all 3 strains were transcribed by strain IbAr10200 replication machinery (NP and L) at approximately the same efficiency in transfected cells, as signal levels (Fig 3A) and titers of tecVLPs produced in BSR-T7 cells (Fig 3B) were similar regardless of the NCR in the minigenome. The differences in signal produced by SW-13 cells incubated with tecVLPs with different NCRs were minor and not statistically significant (p > 0.05, Fig 3C).

### GPC diversity plays a significant role in tecVLP NanoLuc signal strength

As M segments, and therefore GPCs, are commonly exchanged between CCHFV strains [20,21,28–30], we assessed the compatibility between IbAr10200 replication machinery and mature glycoproteins from several pathogenic strains of CCHFV. To study the production of tecVLPs by the IbAr10200 replication machinery, GPCs from CCHFV strains Turkey, Oman, and Afg09 were expressed in the tecVLP system in place of IbAr10200 GPC. The full-length sequences of strain Turkey and Oman GPCs were elucidated prior to use in the tecVLP system. The GPC of the commonly used CCHFV strain IbAr10200 phylogenetically clusters with African CCHFV strains, while Turkey, Oman, and Afg09 GPCs cluster in different nodes of the phylogenetic tree (S2 Fig). Based on amino acid sequences of the entire GPC, these strains were 14–20% different from IbAr10200 GPC (86%, 83%, and 80% amino acid identity between IbAr10200 and Afg09, Oman, and Turkey GPCs, respectively).

**Fig 4:**
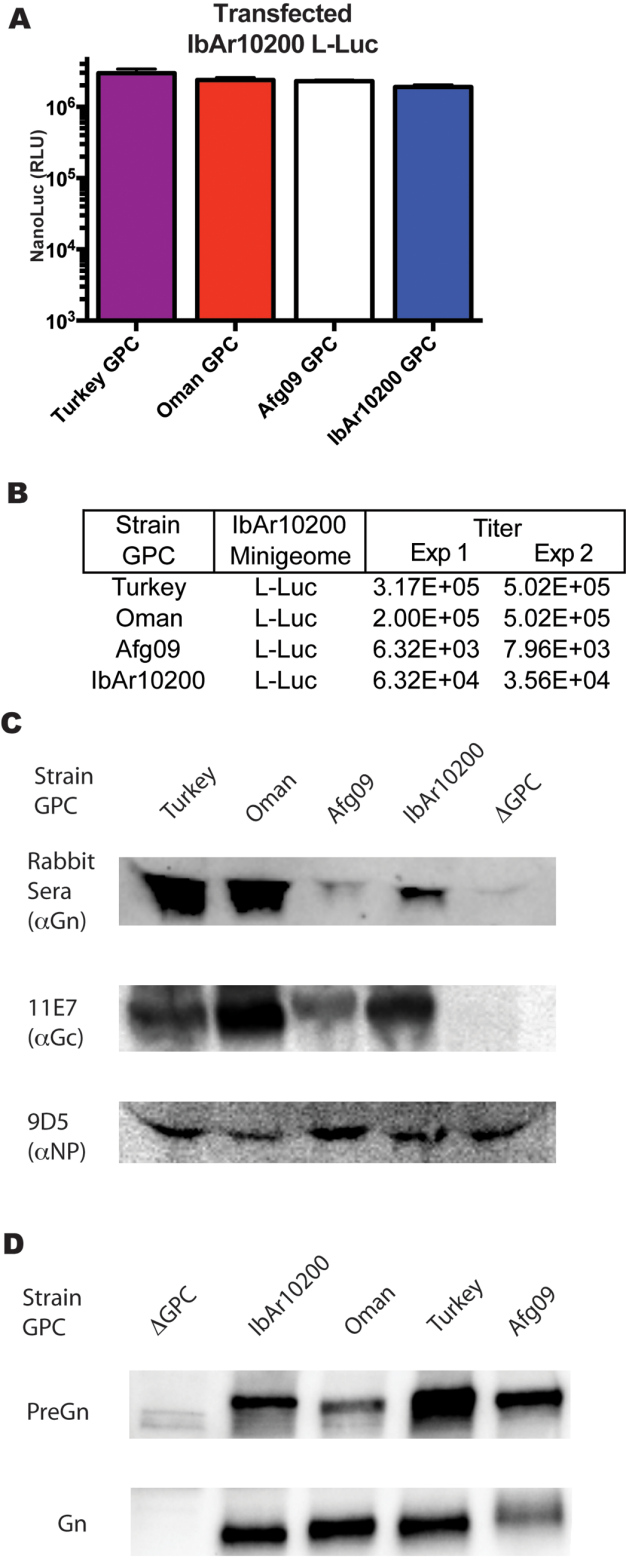
Effects of GPCs from various CCHFV strains on minigenome activity and transcription and tecVLP titers. (A) Absolute NanoLuc signal (in RLU) in BSR-T7 cells transfected with L NCR minigenome and plasmids encoding GPC from Turkey, Oman, Afg09, or IbAr10200 strains. Data are reported as standard error of the mean (average of 2 experiments). (B) tecVLP titers determined by TCID_50_ in SW-13 cells treated with supernatants from BSR-T7 cells in (A). Shown are titers from 2 tecVLP generation experiments. (C) Western blot of CCHFV proteins in concentrated tecVLP supernatants produced by BSR-T7 cells from (A) (ΔGPC obtained from cells transfected with NP and L helper plasmids only). (D) Western blot of Gn precursor and mature Gn produced in cell lysates of transfected BSR-T7 cells. CCHFV proteins were detected by antibodies against IbAr10200 Gn, Gc, or NP, as indicated.

Using GPCs from these strains in transfected cells did not considerably affect NanoLuc signal levels in transfected BSR-T7 cells (Fig 4A), but did affect the amounts of tecVLPs produced. Turkey and Oman strain GPCs led to the highest tecVLP titers, followed by IbAr10200 and Afg09 GPCs (Fig 4B). Only the mature forms of Gn and Gc were detected by western blotting in the cell supernatants containing the tecVLP (~35 kDa for Gn and ~70kDa for Gc, Fig 4C). Levels of Gn were highest when using Turkey and Oman GPCs, lower when using IbAr10200 GPC, and lowest when using Afg09 GPC.

Mature Gn is synthesized from a larger precursor that requires post-translational processing. We tested if the ratios of Gn precursor to mature Gn were equivalent in BSR-T7 cell transfected with GPCs of different CCHFV strains. Western blotting of the cell lysates showed a lower proportion of Afg09 mature Gn over Gn precursor in comparison to other GPCs (Fig 4D).

### GPCs from different CCHFV strains do not affect incorporation of NCRs into tecVLPs

**Fig 5:**
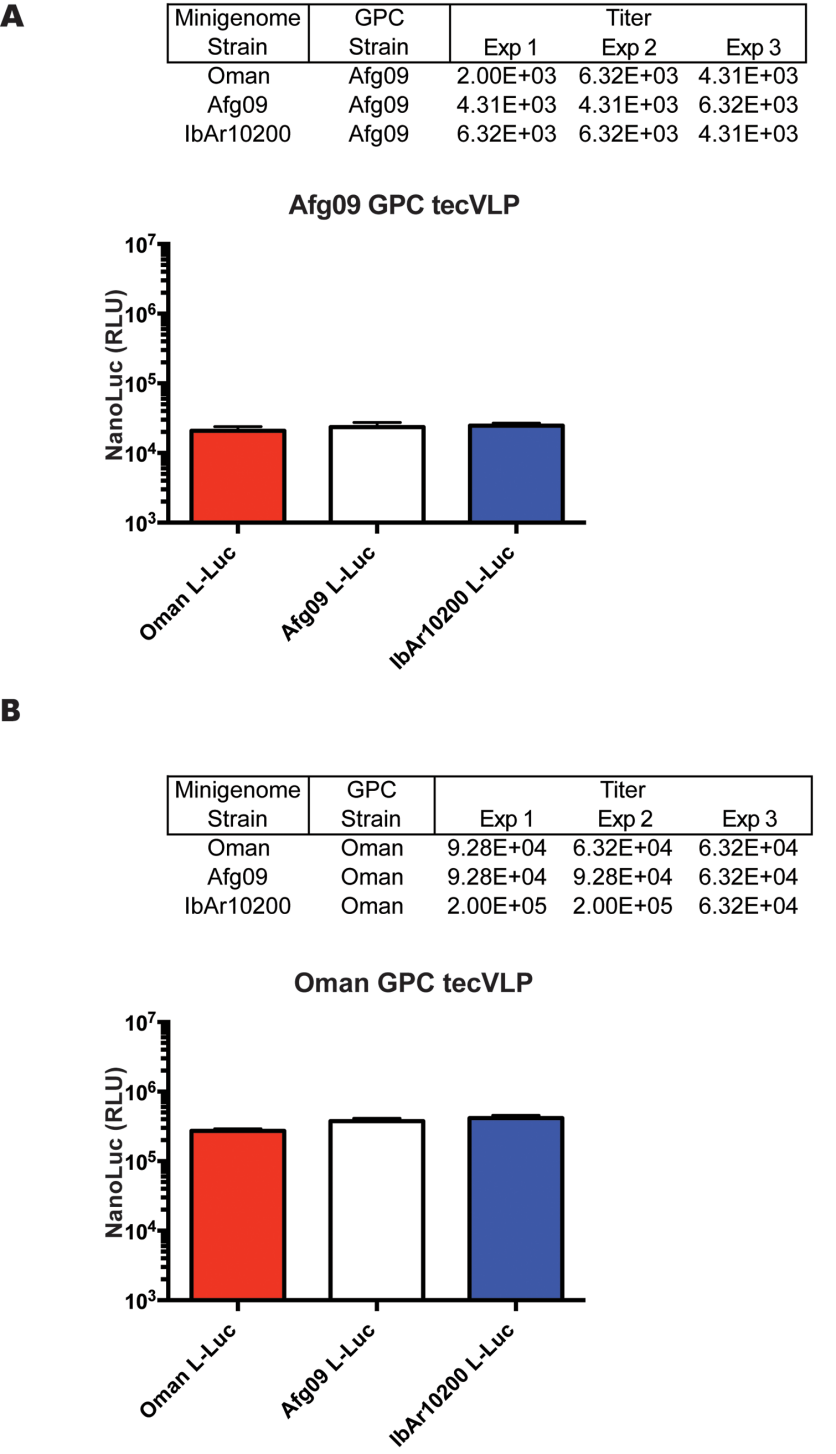
Exchanging surface glycoproteins has a greater impact on tecVLP titer and NanoLuc signal than exchanging minigenomes. (A) TCID_50_ titers/mL and NanoLuc signals in relative light units (RLU) measured in SW-13 cells treated with tecVLPs with Afg09 GPC and with L segment NCR minigenomes from indicated CCHFV strains. NanoLuc data are presented as standard error of the mean (average of 3 experiments), and results of 3 TCID_50_ experiments are shown. (B) TCID_50_ titers/mL and NanoLuc signals measured in SW-13 cells treated with tecVLPs with Oman GPC and L segment NCR minigenomes from indicated CCHFV strains. NanoLuc signal data in tecVLP-treated cells are presented as absolute RLU values which are calculated as signal in SW-13 cells treated with entry-competent VLPs (i.e., containing NP, L, and GPC) minus signal in SW-13 cells treated with VLPs containing only NP, L and the corresponding minigenome. NanoLuc data are reported as standard error of the mean (average of 3 experiments), and results of 3 TCID_50_ experiments are shown.

Due to a previous report that mature CCHFV glycoproteins interact with the NCR regions of the CCHFV genome [17], we used the tecVLP system to address whether this interaction affects the efficiency of tecVLP release in a strain-specific manner. The results demonstrated that while altering the GPC resulted in a substantial difference in tecVLP titer and signal strength, changing minigenome NCRs had a minimal effect on tecVLP signal strength (Fig 5). In addition, using the minigenome NCR and GPC from the same strain together did not affect tecVLP production or NanoLuc signals synergistically; Afg09 GPC did not preferentially increase the signal or titer of Afg09 minigenome tecVLPs (Fig 5A), nor did the Oman GPC preferentially increase the signal of Oman minigenome tecVLPs (Fig 5B) compared to the minigenome containing NCRs from other strains.

### tecVLPs with mature glycoproteins derived from GPCs of pathogenic CCHFV strains efficiently enter a wider range of cell lines

**Fig 6:**
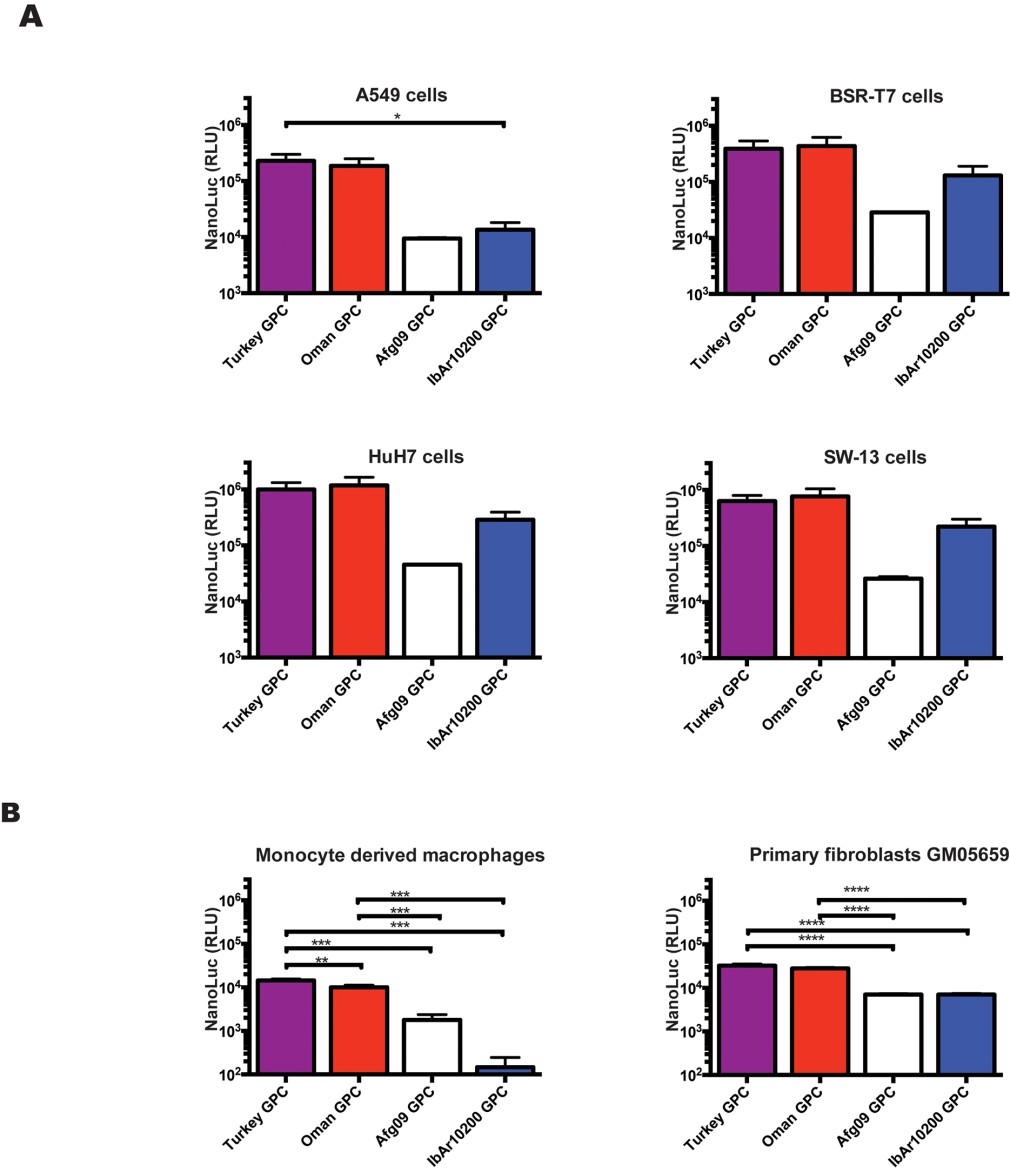
tecVLPs are capable of entering into several immortalized cell lines and primary cells. (A) NanoLuc signal in relative light units (RLU) in indicated cell lines treated with CCHFV tecVLPs containing GPC from several CCHFV strains (average of 3 experiments for each cell line). (B) NanoLuc signal in indicated primary cells treated with tecVLPs containing GPC from various CCHFV strains (average of 4 experiments for each cell line). NanoLuc signal data in VLP-treated cells are presented as absolute RLU values which are calculated as signal in SW-13 cells treated with entry-competent VLPs (i.e., containing NP, L, and GPC) minus signal in SW-13 cells treated with VLPs containing only NP and L. NanoLuc data are reported as standard error of the mean. * p < 0.05, **p < 0.01, ***p < 0.001, ****p < 0.0001.

In order to study the effects of GPC from different CCHFV strains on cell entry in the absence of confounding factors like differences in NP or L, we compared NanoLuc signal levels produced in immortalized and primary cells incubated with tecVLPs containing GPC from several CCHFV strains. tecVLPs containing IbAr10200, Turkey, Oman, or Afg09 GPCs were capable of entering several immortalized cell lines (Fig 6A). Predominantly, the strength of the resulting signal corresponded to tecVLP titers (Fig 4B), with Turkey and Oman GPC tecVLPs yielding the highest titers and signal levels, IbAr1200 GPC producing intermediate levels, and Afg09 GPC tecVLPs yielding the lowest titers and signal levels (compare Figs 4B with 6A). However, NanoLuc signal generated by IbAr10200 GPC-containing tecVLP in A549 cells was on par with Afg09 GPC-containing tecVLPs. Furthermore, when primary human cells were used instead of immortalized cell lines, tecVLPs containing IbAr10200 GPC generated less signal than tecVLPs containing Afg09 GPC; this was especially clear in monocyte-derived macrophages, which seemed refractory to entry by tecVLPs with IbAr10200 GPC (Fig 6B).

### tecVLPs are selectively inhibited by antiviral drugs and mAbs

Efforts to screen CCHFV inhibitors have been hindered in part by the lack of high-throughput quantitative molecular systems that can be used in a BSL-2 setting. In order to overcome this limitation, we evaluated the potential of the tecVLP system to be used to screen compounds and antibodies in a high-throughput, 96-well format. We screened reported inhibitors of CCHFV cell entry (mAbs and chloroquine) and of viral transcription (ribavirin) using SW-13 cells treated with tecVLPs expressing glycoproteins from several CCHFV strains.

**Fig 7:**
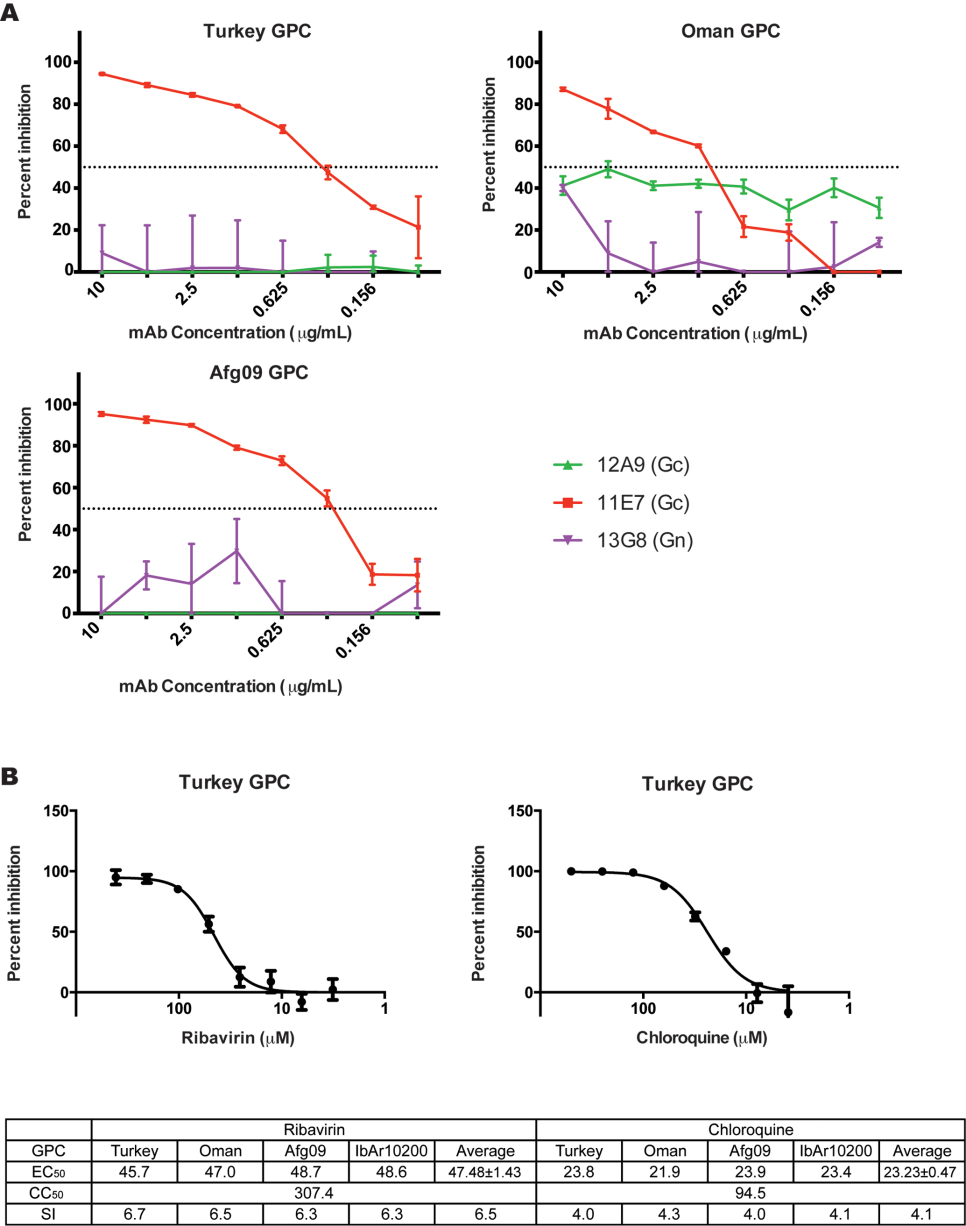
Inhibitors of CCHFV entry and transcription inhibit tecVLP signal production. (A) Neutralization assays of tecVLPs treated with mAbs. Percent inhibition was calculated by comparing the NanoLuc signal strength in SW-13 cells treated with tecVLP and mAbs to signal in cells treated with tecVLPs and a control mAb. (B) Percent inhibition (compared to untreated tecVLP) in SW-13 cells treated with tecVLPs and indicated inhibitors (top panel), and 50% effective concentration (EC_50_), 50% cytotoxicity concentration (CC_50_), and selectivity index (SI) for ribavirin and chloroquine in these cells (bottom panel, average of 3 experiments).

The non-neutralizing mAb 13G8 (targeting IbAr10200 PreGn; Fig 2B) and the neutralizing mAb 12A9 (targeting IbAr10200 Gc; Fig 2B) did not neutralize tecVLPs containing Turkey, Oman, or Afg09 GPCs. However, the neutralizing mAb 11E7 (targeting the IbAr10200 Gc) effectively neutralized all tecVLPs (Fig 7A). This demonstrates that the tecVLP system can be used to differentiate between strain-specific and broadly acting neutralizing mAbs and to rapidly assess mAb effectiveness.

While mAbs are expected to bind different CCHFV glycoproteins with varying efficacy, small molecule CCHFV inhibitors should have similar inhibitory effects regardless of the GPC used. Indeed, using chloroquine or ribavirin on tecVLP-treated SW-13 cells decreased the signal produced by tecVLPs with equal efficiency regardless of the GPC constructs used to produce tecVLPs (Fig 7B). The average EC_50_ was 23.23 ± 0.47 μM for chloroquine and 47.48 ± 1.43 μM for ribavirin, which is consistent with previously reported values [31–33]. The CC_50_ of chloroquine and ribavirin were 94.58 μM and 307.4 μM, respectively, with a corresponding average SI of 4.1 and 6.5, respectively (Fig 7B). These data suggest that the tecVLP system may be effective in screening inhibitors in a medium- or high-throughput set-up.

## Discussion

CCHF is a geographically widespread, life-threatening illness characterized by severe flu-like and hemorrhagic symptoms and relatively high case fatality rates. Research on CCHFV, the causative agent of CCHF, has been hampered by a number of factors. Requirement for high-containment laboratories, limited strain availability, and lack of robust molecular tools for studying CCHFV all stalled basic CCHFV research. The development of VLP systems, especially those that can be simply scaled as needed, and that allow the study of CCHFV in a safe and effective manner, is a major step towards accelerating basic and applied research on this important public health risk.

While our work builds on a previously reported minigenome system [12] and is not the first VLP system for CCHFV to be documented [13], our system possesses several important advantages. (1) We demonstrated that the generated tecVLPs are morphologically consistent with CCHFV, and cell entry was neutralized by same monoclonal antibodies as authentic virus (Fig 2) [27]. (2) A high yield of tecVLPs may be generated in 3 days without passaging in transfected cells (Fig 1C). (3) Recipient cells may be used without resource-consuming pre-treatment steps, such as transfection of helper plasmids (Fig 4); (4) therefore, the system reflects primary transcription/replication in a more natural setting than plasmid overexpression, and (5) a wider range of cell lines may be used (Fig 5). (6) GPCs and minigenomes from divergent CCHFV strains may be used (Figs 3, 4 and 6), expanding the utility of this system. Importantly, the tecVLP system is safe for using outside of high-containment laboratories, since it does not generate infectious virus (S1 Fig). Due to the lack of tecVLP replication, experiments assessing strain virulence cannot be conducted in vivo, but tecVLPs can likely be used to investigate the underlying differences between pathogenic and non-pathogenic or weakly pathogenic strains. Therefore, this system has the potential to greatly aid the study of CCHFV particle assembly, egress, entry, and primary transcription/replication in vitro and in vivo. In addition, due to ease of scaling, the tecVLP system may be suitable for high-throughput screens of antiviral agents.

To assess the usefulness of our system, we examined several aspects of CCHFV biology, and tested the tecVLP system as a screening tool for CCHFV inhibitors. Based on previous minigenome data [12,13], we hypothesized that CCHFV replication machinery preferentially transcribes, replicates, and packages the CCHFV L genomic segment over other segments. While L segment NCRs sometimes produced higher NanoLuc signals in transfected cells, as in previous reports [12,13], the amounts of tecVLPs produced did not substantially differ between minigenomes (Fig 1). In order to be consistent between experiments, however, we used the L NCR-based minigenomes and tecVLPs in most of our experiments.

Our first sets of experiments focused on observing the ability of IbAr10200 replication machinery to replicate minigenomes possessing NCRs of 2 pathogenic CCHFV strains. While the termini of the NCRs are highly conserved (1–2 differences in the terminal 30 nucleotides), significant differences are seen in the internal regions of the NCRs (S3 Fig). Thus, we hypothesized that the NCR sequences that differed may affect transcription, replication, and/or packaging efficiency by the strain IbAr10200 replication machinery. However, we saw no substantial differences in reporter signal or tecVLP production (Fig 3). While the difference was small, we were surprised to find that the IbAr10200 replication machinery was not best adapted to transcribe its own minigenome compared to minigenomes from divergent strains. Therefore, we concluded that CCHFV IbAr10200 replication machinery efficiently incorporates L NCRs from several CCHFV strains into tecVLPs, probably because NCRs do not differ sufficiently at the sites critical for transcription, replication, and packaging. This finding may have significant implications for assessing reassortment potential between CCHFV strains, as it suggests that CCHFV strains have evolved to easily exchange segments during co-infections. Indeed, epidemiological studies have frequently reported genetic reassortment between CCHFV strains, especially in the GPC-encoding M segment [20,21,30,34].

Due to high reported reassortment frequency and experimental reports from other bunyaviruses [35–37], we also examined the interaction between the IbAr10200 replication machinery and GPCs from other CCHFV strains by determining efficiency of tecVLP production using these constructs. We were surprised to see that GPC from Afg09, the strain phylogenetically most closely related to IbAr10200, resulted in lower tecVLPs titers than IbAr10200, while the more distantly related Oman and Turkey GPCs resulted in higher tecVLP titers than IbAr10200 GPC (Fig 4B and S2 Fig). While all GPCs were transcribed, translated, and secreted into the cell supernatant, the amounts of mature Gn and Gc generally correlated with tecVLP titers (Turkey ≈ Oman > IbAr10200 > Afg09; Fig 4C). Cell lysates of transfected BSR-T7 cells showed that while relatively high levels of the Gn precursor are produced for all GPCs, the Afg09 strain Gn precursor appeared to be inefficiently processed (Fig 4D). These findings suggest that post-translational cellular glycoprotein processing is likely responsible for the amount of tecVLP produced. In addition, we found that tecVLPs containing different GPCs behaved similarly in several immortalized cell lines, with Turkey and Oman GPC resulting in highest signal levels, followed by IbAr10200, and Afg09 GPC resulting in the lowest signal levels (Fig 6A). However, in A549 cells, primary human fibroblasts, and especially primary human monocyte-derived macrophages, IbAr10200 GPC-containing tecVLPs generated lower signal levels than tecVLP with other GPC strains (Fig 6B). As these tecVLPs differed only in the surface glycoproteins, the ability to enter cells is likely to be the cause of different signal levels (Fig 6). This finding shows that in addition to efficiently incorporating into the viral particle, mature glycoproteins must facilitate entry into multiple cell types. Furthermore, these data suggest that the IbAr10200 GPC is “lab-adapted,” probably due to multiple passages, and has poorer entry capacity than proven pathogenic CCHFV strains, which enter cells more efficiently. Thus, IbAr10200 may not accurately represent the mechanisms of entry used by highly pathogenic CCHFV strains. Additionally, since pathogenic CCHFV strains do not necessarily produce higher tecVLP titers than IbAr10200 but do facilitate efficient entry into immune cells, our data suggest that the ability to efficiently enter immune cells may be a better assessment of strain virulence than absolute tecVLP production.

Finally, due to the proposed “matrix protein” function of the CCHFV glycoproteins [17], we also tested for any synergy between GPCs and NCR minigenomes from the same CCHFV strain during tecVLP production. We found that, in our system, using the GPC and NCR from the same strain had no synergistic effect on tecVLP production (Fig 5).

Overall our data were unexpected, as we found that while the IbAr10200 replication machinery certainly yields a higher NanoLuc signal when used with certain glycoproteins or minigenomes, it does not always prefer its own glycoprotein or minigenome.

In order to show that our system can be used to screen inhibitors of cell entry and transcription/replication, we used known inhibitors of CCHFV receptor-mediated entry (neutralizing mAbs), endosome-mediated entry (chloroquine), and transcription (ribavirin) on tecVLPs with different glycoproteins. To demonstrate that tecVLPs could be used to identify even relatively minor differences in mAb affinity, we tested several previously reported mAbs, both neutralizing and non-neutralizing, in our assay. The results show that mAbs 11E7 and 12A9, which target Gc and which were previously reported to neutralize IbAr10200, readily neutralize tecVLPs expressing IbAr10200 GPC, while non-neutralizing anti-PreGn and anti-NP mAbs (13G8 and 9D5, respectively) do not neutralize IbAr10200 GPC-containing tecVLPs [27] (Fig 2B). In contrast, tecVLPs containing GPC from other strains are not neutralized by 12A9, but are neutralized by 11E7, identifying 11E7 as a broadly neutralizing anti-CCHFV mAb (Fig 7A). These results both illustrate the power of the tecVLP system to quickly screen mAbs, and show that this system can detect mAbs targeting mature glycoproteins of different stains.

Unlike antibodies, inhibitors targeting common components of tecVLPs or cell entry pathways should behave similarly in all tecVLPs regardless of GPC origin. Indeed, chloroquine (which inhibits viral cell entry) and ribavirin (which inhibits RNA transcription) inhibited all tecVLPs, and both inhibitors had a nearly identical EC_50_ regardless of the GPC used to generate each tecVLP (Fig 7B). Furthermore, the EC_50_ values calculated for the tecVLPs were similar to those reported in previous studies using live CCHFV [31,32]. These data suggest that tecVLPs might be useful as a replacement of live CCHFV in inhibitor screens, allowing high-throughput studies at BSL-2 conditions.

In conclusion, our tecVLP system is a safe, robust, and effective way to study the molecular life cycle of CCHFV. The key features of this system are that it can be used in a regular laboratory with multiple cell types, and can be easily modified to fit the needs of the researcher. We used the system to explore glycoprotein and genome incorporation into tecVLPs, and our findings suggest that compatibility between the glycoprotein and the replication machinery impacts the reassortment potential between CCHFV strains. Furthermore, our study also suggests that tecVLPs may be able to replace live CCHFV in screening both immunological and small molecule inhibitors and evaluating these inhibitors in vitro, both in high- and low-throughput settings.

## Supporting Information

S1 Fig Safety testing of CCHFV tecVLPs. Representative light microscopy (top panels in each set) and confocal microscopy (bottom panels) images of SW-13 cells inoculated with (A) infectious CCHFV, (B) supernatants from BSR-T7 cells producing tecVLPs, or (C) supernatants from untreated BSR-T7 cells (negative control). Following 2 days of incubation, SW-13 cells were stained by standard immunofluorescent assay using CCHFV hyperimmune mouse ascetic fluid (HMAF) or CCHFV PreGn-specific mAb 13G8 as the primary antibody, and with goat anti-mouse Alexa Fluor 488-labeled polyclonal sera as the secondary antibody. Background staining was assessed by incubating SW-13 cells with goat anti-mouse Alexa Fluor 488-labeled polyclonal sera without a primary antibody. (D) NanoLuc signal in relative light units (RLU) in SW-13 cells treated with tecVLP supernatants (p1) or supernatants of SW-13 cells treated with tecVLP supernatants (p2; n = 8 from 1 experiment). Data are reported as absolute NanoLuc signal. All data are reported as standard error of the mean.
(TIF)

S2 Fig Phylogenetic analysis of amino acid sequences of CCHFV glycoproteins. Complete amino acid sequences of GPC proteins from various CCHFV strains were compared by maximum likelihood analysis. Sequences were created during the course of this experiment (Turkey and Oman) or obtained from GenBank (Afg09). Bootstrap support values are indicated at the nodes, and accession numbers are indicated. Highlighted in grey are the GPCs sequenced during the course of these studies. Black frames outline the GPCs used in the studies.
(TIF)

S3 Fig Alignment of the L non-coding regions (NCRs) of CCHFV strains IbAr10200, Afg09, and Oman. Sequences were derived from accession numbers AY389508, HM452307, and DQ211619, for IbAr10200, Afg09, and Oman NCRs, respectively. Terminal 30 nucleotides at both 5′ and 3′ RNA ends are highlighted in grey.
(TIF)
