# Supplementary figures and images for: Assessment of Inhibitors of Pathogenic Crimean-Congo Hemorrhagic Fever Virus Strains Using Virus-Like Particles
Source: PLoS Negl Trop Dis. 2015 Dec 1;9(12):e0004259. doi: 10.1371/journal.pntd.0004259 (PMC4666410; doi:10.1371/journal.pntd.0004259)

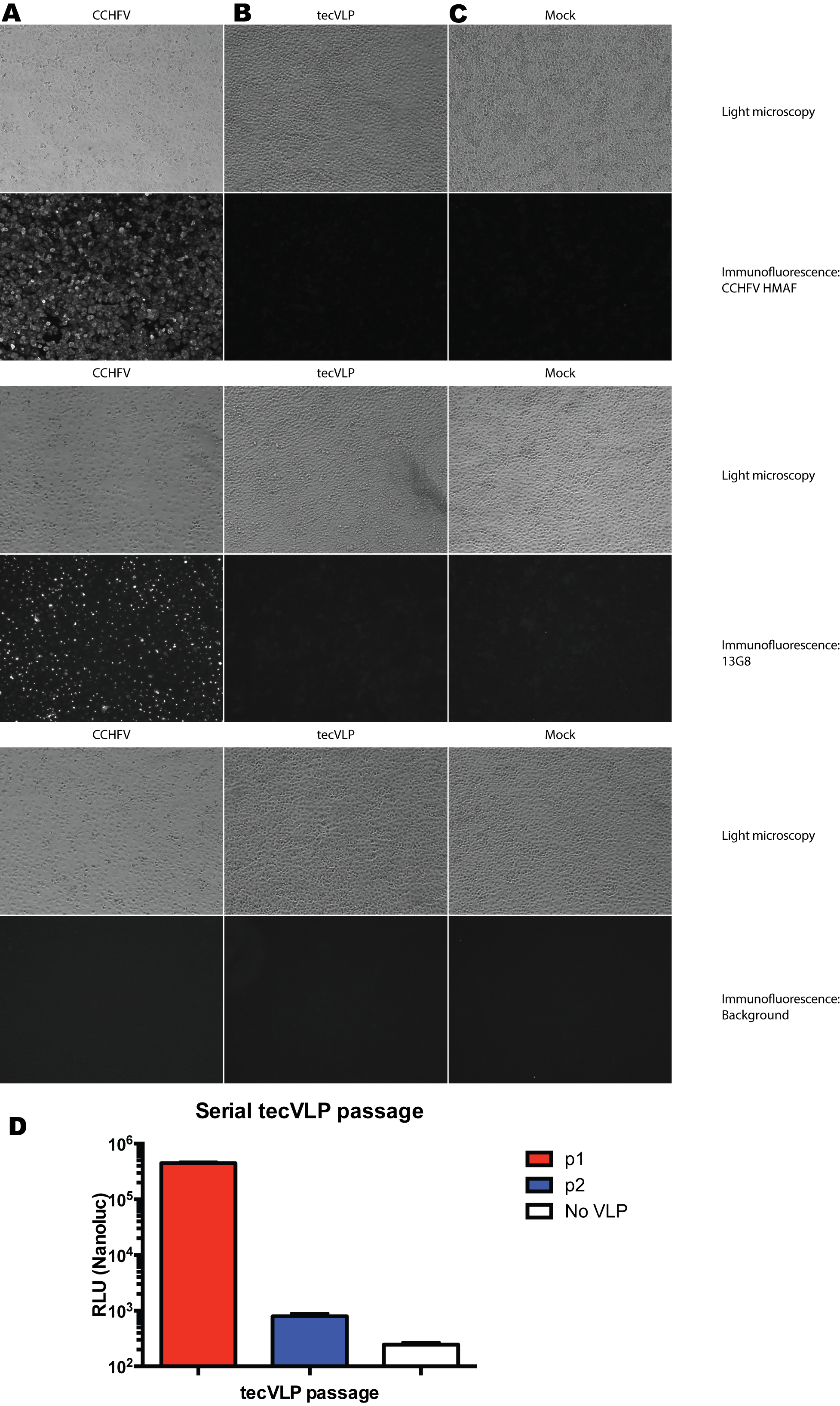

Supplement: S1 Fig — Representative light microscopy (top panels in each set) and confocal microscopy (bottom panels) images of SW-13 cells inoculated with (A) infectious CCHFV, (B) supernatants from BSR-T7 cells producing tecVLPs, or (C) supernatants from untreated BSR-T7 cells (negative control). Following 2 days of incubation, SW-13 cells were stained by standard immunofluorescent assay using CCHFV hyperimmune mouse ascetic fluid (HMAF) or CCHFV PreGn-specific mAb 13G8 as the primary antibody, and with goat anti-mouse Alexa Fluor 488-labeled polyclonal sera as the secondary antibody. Background staining was assessed by incubating SW-13 cells with goat anti-mouse Alexa Fluor 488-labeled polyclonal sera without a primary antibody. (D) NanoLuc signal in relative light units (RLU) in SW-13 cells treated with tecVLP supernatants (p1) or supernatants of SW-13 cells treated with tecVLP supernatants (p2; n = 8 from 1 experiment). Data are reported as absolute NanoLuc signal. All data are reported as standard error of the mean. (TIF) [file pntd.0004259.s001.tif]

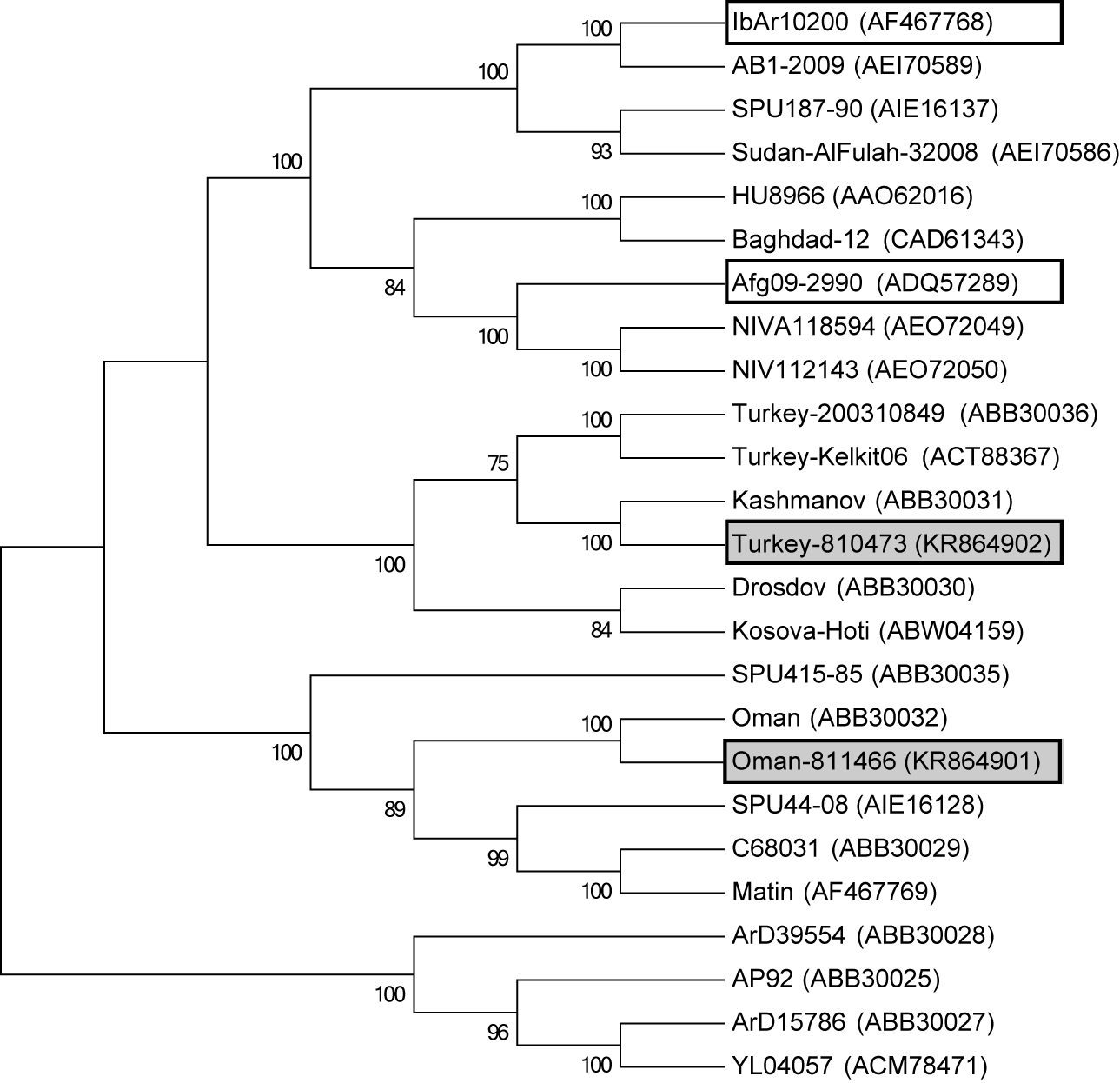

Supplement: S2 Fig — Complete amino acid sequences of GPC proteins from various CCHFV strains were compared by maximum likelihood analysis. Sequences were created during the course of this experiment (Turkey and Oman) or obtained from GenBank (Afg09). Bootstrap support values are indicated at the nodes, and accession numbers are indicated. Highlighted in grey are the GPCs sequenced during the course of these studies. Black frames outline the GPCs used in the studies. (TIF) [file pntd.0004259.s002.tif]

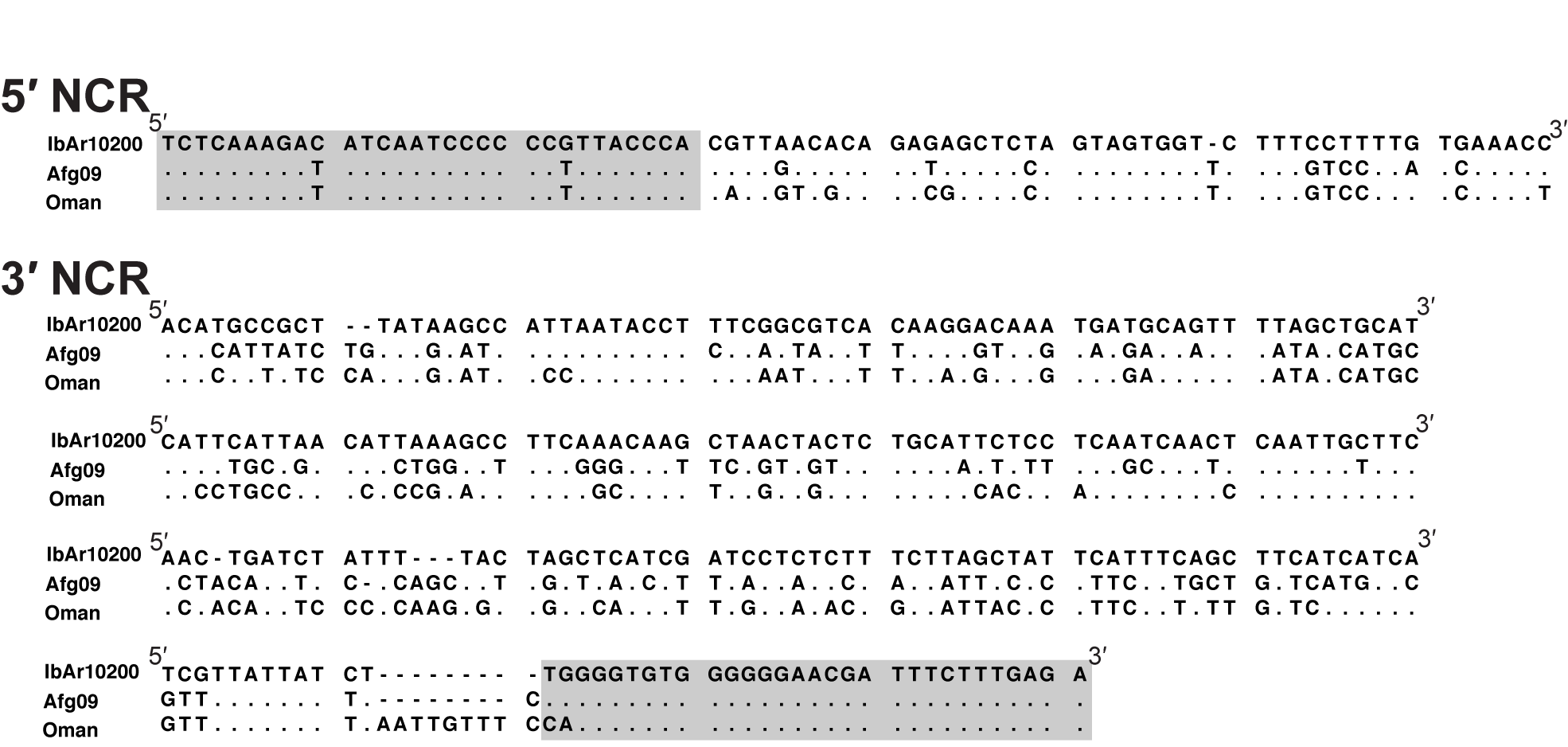

Supplement: S3 Fig — Sequences were derived from accession numbers AY389508, HM452307, and DQ211619, for IbAr10200, Afg09, and Oman NCRs, respectively. Terminal 30 nucleotides at both 5′ and 3′ RNA ends are highlighted in grey. (TIF) [file pntd.0004259.s003.tif]
